# Interdisciplinary groups perform better than intradisciplinary groups in online group discussion activities

**DOI:** 10.1080/10872981.2021.1886649

**Published:** 2021-02-09

**Authors:** Taro Yamashita, Shuji Osawa, Kunio Ota, Takahiro Minami, Yuma Morisaki, Yutaro Takahashi, Tomoya Itatani, Satoshi Hara, Toshikatsu Tamai, Makoto Fujiu, Hideki Nomura, Rie Okamoto

**Affiliations:** a Department of General Medicine, Kanazawa University Hospital, Kanazawa, Japan; b Division of Environmental Engineering Design, Graduate School of Natural Science and Technology, Kanazawa, Japan; c Medical Education Research Center, Graduate School of Medical Sciences, Kanazawa, Japan; d Division of Health Sciences, Graduate School of Pharmaceutical Science, Kanazawa University, Kanazawa, Japan

**Keywords:** Interprofessional education, online group work, decision making, professional hierarchy, undergraduate medical education

## Abstract

Online classes have been provided for health-care pre-licensure learners during the novel coronavirus disease 2019 pandemic. The purpose of this study was to evaluate the utility of online group work in interprofessional education. A total of 209 students were assigned to 50 groups (18 medical student groups, 13 nursing student groups, and 19 mixed medical/nursing student groups). Learners performed group work during the orientation for the course, which was hosted using an online video conferencing system. The learners first performed the activity individually (10 min) and then engaged in a group discussion to reach consensus on their answers (30 min). We calculated the scores before and after the group discussion and shared the results with the students. Scores were improved after the group discussion (mean ± SEM, 23.7 ± 0.9) compared with before (37.3 ± 1.3) (P < .0001). Lower scores after the group discussion, which indicated the effect of the group discussion on making better decisions, were observed most in the mixed medical/nursing student groups, followed by the nursing student and medical student groups. We noted only 3 groups in which the group discussion showed a negative effect on decision-making: all 3 of these groups were mixed (3 of 19 groups; 16%). These data demonstrated the power of group discussion for solving tasks when the participants’ professional fields were mixed. However, the small size of the interdisciplinary groups might have resulted in less effective discussion, which might be due in part to psychological barriers arising from professional differences. Online group work is effective for facilitating discussion and building consensus about decisions in interprofessional education for medical and nursing students. Potential psychological barriers may exist in about 16% of mixed group students at the start, which should be kept in mind by instructors.

**Abbreviations**: COVID-19: coronavirus disease 2019; IPE: Interprofessional Education; NASA: National Aeronautics and Space Administration; SD: standard deviation; WHO: World Health Organization

## Introduction

The importance of interprofessional collaboration has become increasingly evident in light of the multiple comorbidities in elderly patients and the complexity of the health care system [1]. Miscommunication and lack of coordination have the potential to negatively impact patient outcomes. Meanwhile, a multidisciplinary approach may reduce the cost of medical care and facilitate the safe and effective management of patients with chronic and complex diseases [2,3]. Collaboration among health care providers needs to be optimized for each patient, and thus interprofessional education (IPE) and collaboration are considered to play a fundamental role in improving health care outcomes [2]. Accordingly, the World Health Organization (WHO) published a paper entitled ‘Framework for Action on Interprofessional Education and Collaborative Practice’ [4] which emphasized the importance of developing and integrating IPE in health care education curricula.

IPE curricula based on the WHO framework have been introduced for medical and nursing students to learn how to collaborate effectively to improve health care outcomes in the community. Although there is no consensus about the timing of integrating IPE into curricula for health-care pre-licensure learners [5], Kanazawa University recently introduced IPE training courses for second- and fourth-year medical and nursing students, which they must complete before starting their clinical clerkships. Despite the importance in the IPE curricula of building a collaborative practice environment [6], it is unclear whether the introduced IPE training is effective at developing communication skills that facilitate collaboration.

Here we quantitatively evaluated the degree of collaborative practice by having medical and nursing students engage in a group work exercise before they started the assignments for their respective IPE curricula. Due to the recent novel coronavirus disease 2019 (COVID-19) pandemic, we switched from in-person IPE training to online group work. To ensure that all students could perform the online communication and discussion assignments effectively, we introduced a group discussion activity, which involved ranking a list of items necessary for survival on the moon in order of importance, during the class orientation.

## Methods

### Participants

All 213 fourth-year students were requested to participate in the IPE class orientation, and 209 (98%) participated. Of the 121 medical students, 21 (17%) were female, and of the 88 nursing students, 82 (93%) were female. The students were randomly assigned to 50 different groups consisting of 4–5 students each: 18 groups consisted of only medical students, 13 groups consisted of only nursing students, and 19 groups consisted of a mix of medical and nursing students.

### Online IPE orientation

To develop the online IPE curricula for fourth-year medical and nursing students, a total of 10 professors, associate professors, and assistant professors from the Faculties of Medicine, Nursing, Engineering, and Higher Education Research and Development, participated in an online meeting to discuss how to implement the IPE course during the COVID-19 pandemic. Furthermore, 9 graduate students participated in the meeting and as active learning assistants. We utilized a videoconferencing system (Webex; Cisco Systems, Inc., San Jose, CA) for the online IPE course because our University had obtained the licenses before the start of the COVID-19 pandemic. Because a questionnaire survey of students before the IPE course indicated that a limited number of them had previously taken online classes, we decided to use a group discussion exercise as an ice breaker activity before the start of the course. We selected the activity ‘NASA Exercise: Survival on the Moon’ as a well-documented scenario-based exercise to practice the communication and discussion skills required for the online IPE course [7]. The detailed information is available at https://www.nasa.gov/pdf/166504main_Survival.pdf. We used Webex Meetings (Cisco Systems, Inc., San Jose, CA) to introduce the scenario to the participants.

The exercise involves ranking a list of items necessary for survival on the moon from 1 (most important) to 15 (least important). At first, the students performed the exercise by themselves for 10 min. Then, they discussed their rankings with their assigned groups for 30 min using the videoconferencing system. All groups were instructed to reach a consensus on the ranking of items [8]. All students remained in their allocated groups throughout the exercise. Student fidelity was checked by visually confirming their presence in the Webex videoconference window. We used Google Sheets (https://www.google.com/intl/ja_jp/sheets/about/) to record the participants’ scores on the scenario-based exercise.

Exercise scores were calculated by summing the point differentials from the individual and group rankings versus the official NASA ranking. Lower scores indicate better decision-making (i.e., closer to the official NASA ranking), and students were given one of six letter grades based on their score: A, 0–25, excellent; B, 26–32, good; C, 33–45, average; D, 46–55, fair; E, 56–70, poor; F, 71–112, very poor [8]. In addition, students who achieved exercise scores of 15 or less (0–15) were regarded as exceptional in this study. Improvement scores were calculated as the average of the difference between the individual scores before the group discussion and the score of the consensus ranking after the group discussion.

Once the course began, students were asked to complete a questionnaire about the IPE program at the end of every class. In this study, we considered only one of the questionnaire items: ‘Do you think the interprofessional education program will serve to clarify the problems of residents/patients you are responsible for?’ The students could choose from among 5 answers (strongly agree, agree, neither agree nor disagree, disagree, strongly disagree), and we regarded ‘strongly agree’ and ‘agree’ as positive answers.

### Ethics approval and consent to participate

The study was approved by the Institutional Review Board of Kanazawa University (No. 977–1). Consent to participate was obtained through an opt-out process.

### Statistical analysis

Paired *t*-tests were used to evaluate the changes in exercise scores before and after the group discussion. Unpaired *t*-tests were used to evaluate the differences in exercise scores according to sex and professional field. The effect of the group discussion on exercise scores was evaluated using McNemar’s test. These analyses were performed using GraphPad Prism software ver. 8.2.0 (GraphPad Software, San Diego, CA) or SPSS software ver. 23.0 (IBM Japan, Ltd., Tokyo, Japan). Two-sided P values of 0.05 or less were considered statistically significant.

### Availability of data and materials

All data will be made available by the corresponding author upon reasonable request.

## Results

### Web-based group discussion had positive effects on decision making

**Figure 1.**
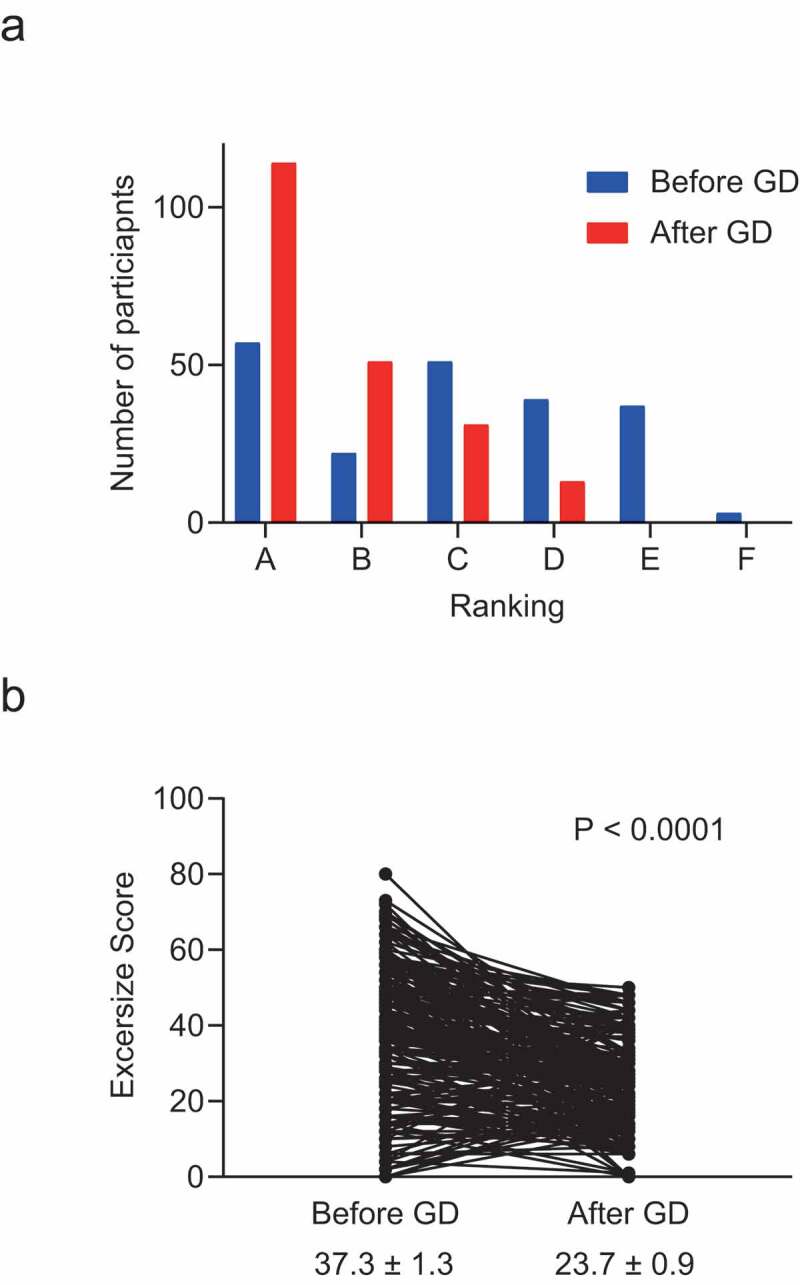
Effect of group discussion on decision making. (a) Histogram of all participants and exercise scores before (blue bars) and after (red bars) group discussion. Grades A, B, C, D, E, and F indicate scores of 0–25 (excellent), 26–32 (good), 33–45 (average), 46–55 (fair), 56–70 (poor), and 71–112 (very poor), respectively. (b) Exercise scores before and after group discussion. The exercise scores were clearly lower after group discussion, indicating improved decision making from the group discussion with statistical significance (paired *t*-tests, P < 0.0001). GD; group discussion

**Figure 2.**
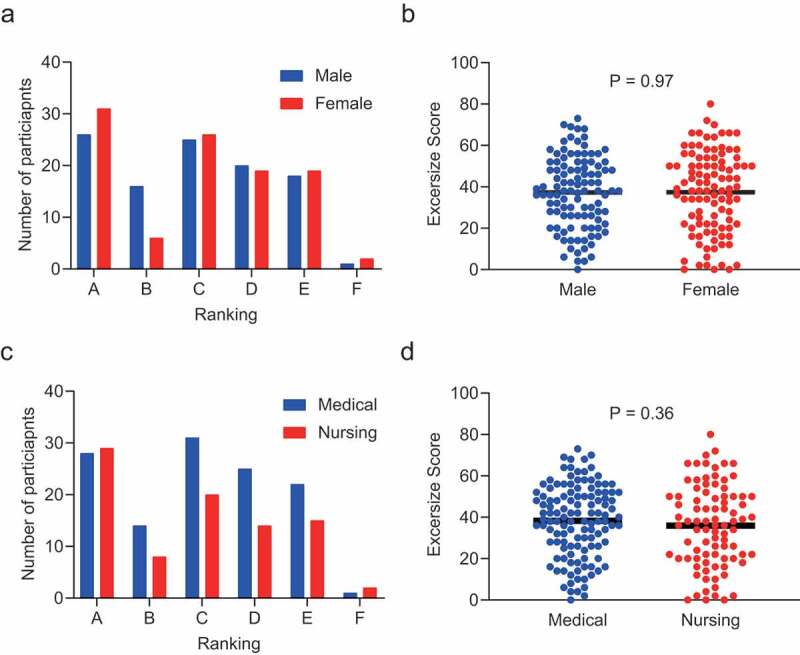
Exercise scores and participants’ professional fields. (a) Histogram of exercise scores before group discussion according to gender. (b) Scatter plots of exercise scores before group discussion according to gender (black bar; mean). No differences were detected between men and women in terms of exercise score distribution (unpaired *t*-tests, P = 0.97). (c) Histogram of exercise scores before group discussion according to professional field. (d) Scatter plots of exercise scores before group discussion according to professional field (black bar; mean). No differences were detected between medical and nursing students in terms of the exercise score distribution (unpaired *t*-tests, P = 0.36)

**Figure 3.**
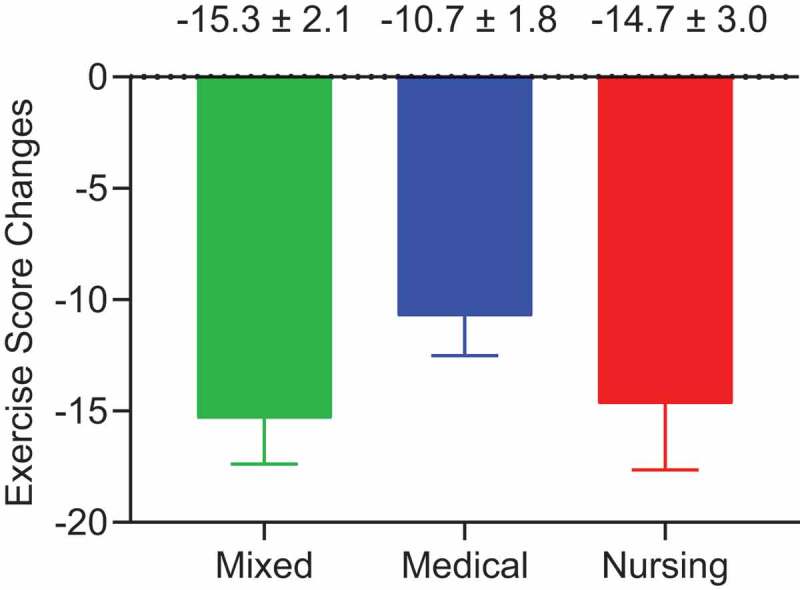
Histogram of exercise score changes before and after group discussion. The greatest improvement in exercise scores after the group discussion was observed in the mixed student groups (−15.3 ± 2.1), followed by the nursing groups (−14.7 ± 3.0) and medical groups (−10.7 ± 1.8)

Of the 213 students enrolled in the IPE course, 209 participated in the online class orientation. All students successfully logged on to the videoconferencing system and were able to access the class material and participate in the group discussion and report their rankings without incident. A histogram of exercise scores before and after the group discussion for all students is shown in Figure 1(a). The number of students who received a grade of A (excellent) or B (good) clearly increased after the group discussion. The exercise scores ranged from 80 to 0 (37.3 ± 1.3) (mean ± SEM) before the group discussion, and from 50 to 0 (23.7 ± 0.9) after, which was statistically significant (P < 0.001, Figure 1(b)). The distribution of scores before the group discussion showed no significant differences according to gender or professional field (Figure 2(a–d)). We evaluated the scores of the medical groups, nursing groups, and mixed groups separately before and after the group discussion and found positive effects of the group discussion in all three groups (Figure 3). The positive effect of group discussion (reduction of exercise scores) was most prominent in the mixed groups (−15.3 ± 2.1), followed by the nursing groups (−14.7 ± 3.0) and the medical groups (−10.7 ± 1.8). We evaluated the frequency of students who achieved excellent scores before and after the group discussion in each group and statistically evaluated the value of group discussion on improved decision-making by McNemar’s test. The effect of group discussion on achieving excellent scores (0–25) was similarly observed in each group with statistical significance (Table 1). In contrast, when we evaluated the effect of group discussion on getting exceptional scores (0–15), the positive effect was observed only in the mixed groups (P < 0.0001); a positive effect was not detected in the medical and nursing groups, which was statistically significant (Table 2). These data indicated that although group discussion had a positive effect on decision-making as a whole, the effect was highest in groups composed of students with different professional fields. Exceptional decision-making was observed most when students with different professional fields were mixed.

**Table 1.** Number of participants who achieved excellent scores (exercise scores ≦ 25) before and after group discussion in mixed, medical, and nursing groups

		Achievement of excellent score after group discussion			
		No	Yes	Total	P value*
*Mixed Group*					
Achievement of excellent score before group discussion	No	29	30	59	
	Yes	8	21	29	
Total		37	51	88	0.001
*Medical Group*					
Achievement of excellent score before group discussion	No	41	18	59	
	Yes	2	10	12	
Total		43	28	71	<0.0001
*Nursing Group*					
Achievement of excellent score before group discussion	No	15	19	34	
	Yes	0	16	16	
Total		15	35	50	<0.0001

*Differences were analyzed by McNemar’s test

**Table 2.** Number of participants who achieved exceptional scores (exercise scores ≤ 15) before and after group discussion in mixed, medical, and nursing groups

		Achievement of exceptional score after group discussion			
		No	Yes	Total	P value*
*Mixed Group*					
Achievement of exceptional score before group discussion	No	48	30	78	
	Yes	6	4	10	
Total		54	34	88	<0.0001
*Medical Group*					
Achievement of exceptional score before group discussion	No	55	8	63	
	Yes	3	5	8	
Total		58	13	71	0.23
*Nursing Group*					
Achievement of exceptional score before group discussion	No	27	13	40	
	Yes	5	5	10	
Total		32	18	50	0.096

*Differences were analyzed by McNemar’s test

### A negative effect of group discussion was observed on decision-making in a subset of mixed medical/nursing student groups

**Figure 4.**
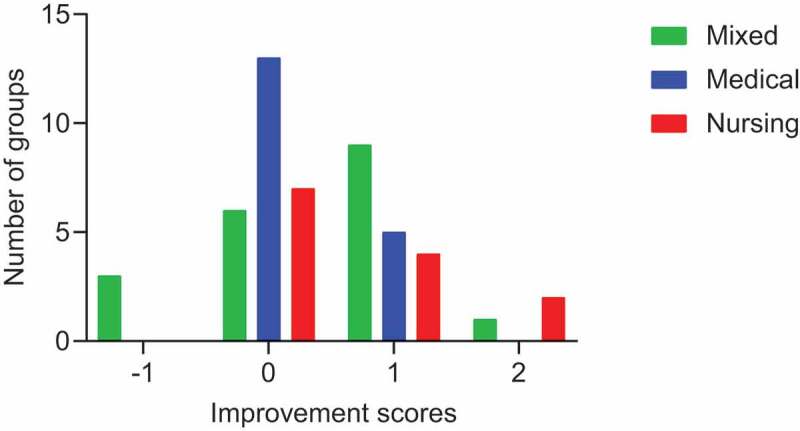
Histogram of all groups and improvement scores. A negative effect of group discussion on decision making was detected only in 3 mixed groups, whereas all other groups showed positive effects of group discussion on decision making

**Figure 5.**
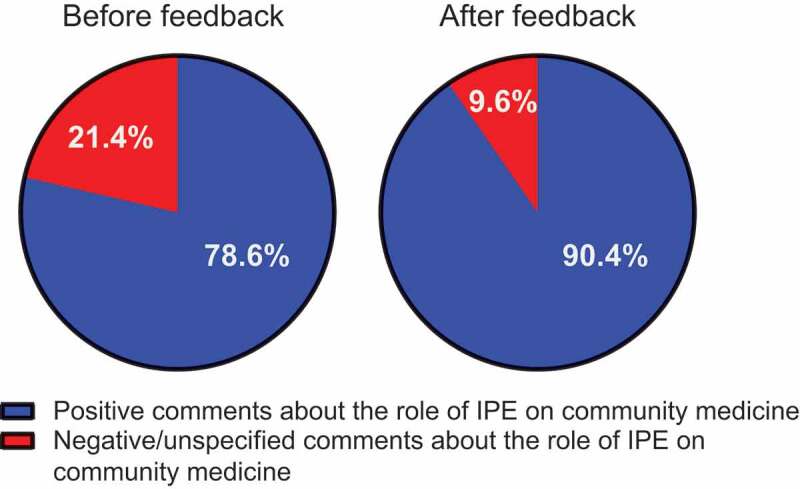
Pie charts illustrating the percentage of the comments about the role of IPE before and after the findings about group discussion outcome were shared with the students. Red indicates negative/unspecified responses to the questionnaire item about the role of IPE. Blue indicates positive comments to the item about the role of IPE

We next evaluated the degree of improvement in exercise scores for the participants in each group before and after the group discussion. We calculated the improvement scores for each group as described in the Methods section and found that 47 of 50 groups showed a clear improvement (Figure 4). However, a negative effect of group discussion was observed in 3 groups, all of which were mixed groups (green bar in Figure 4). We closely examined the individual scores of the students in these groups before and after the group discussion. All 3 groups were composed of 7 medical students and 6 nursing students each. Three of the 7 medical students and 3 of 6 nursing students showed worse exercise scores after the group discussion. Given that this phenomenon was not observed in non-mixed groups, we speculated that interdisciplinary nature of the groups might have led to some hesitation on the part of these students to engage in a frank discussion about their decision-making process. Therefore, we shared our findings with all the students, and then asked them to complete and submit questionnaires at the end of every IPE class. One hundred ninety-one and 208 students answered the questionnaires before and after we shared out findings, respectively. Serial answers were obtained from 187 students, and the questionnaire recovery rate was 90%. We evaluated the effects of sharing our findings with the students on their answers to the item about the IPE program. The number of negative answers (9.6%) was clearly decreased after sharing our findings compared with that (21.4%) before (Figure 5). We evaluated the effects of sharing our findings on the answers to item about the IPE program, and the number of participants who responded positively was clearly increased with statistical significance (P < 0.001) (Table 3).

**Table 3.** Number of participants who answered positively about the role of IPE programs before and after sending the findings of the group discussion outcome were shared

		Comments about the IPE program after the findings were shared			
		Negative/ unspecified	Positive	Total	P value*
Comments about IPE programs before findings were shared	Negative/ unspecified	12	28	40	
	Positive	6	141	147	
Total		18	169	187	<0.0001

*Differences were analyzed by McNemar’s test

## Discussion

During the COVID-19 pandemic, we performed all IPE training for medical and nursing students online to maintain social distancing between students and faculty members. To facilitate productive online group work, we used a group discussion exercise as an ice breaker activity in the IPE class orientation. All students successfully logged on to the videoconferencing system and were able to access the class material and participate in the group discussion without incident. In addition, the questionnaire recovery rate exceeded 90%, which may be related to the ease of completing answers using the online system compared with paper-based methods. Thus, the online IPE training was considered successful and seemed to facilitate the enhancement of group discussion in the IPE course.

We divided the students into 50 groups, which resulted in a relatively small group size of 4–5 students per group. Although the optimal size for strong team performance remains a matter of debate, the widely accepted size is 5 people (https://knowledge.wharton.upenn.edu/article/is-your-team-too-big-too-small-whats-the-right-number-2/). However, it was unclear how team size affected performance in online group work, and our data did not provide any evidence on this point.

This study highlighted the utility of group discussion on better decision. Furthermore, our data clearly indicated the power of group discussion for solving tasks when participants’ professional fields were mixed. Previous reports have indicated the positive effects of IPE on occupational therapy education and on-site training for medical and nursing students [9–11]. It is reasonable to consider that ideas or ways of thinking might be similar when participants are studying in the same professional field. Such similarity might hamper the exchange of the unexpected ideas. We believe that our data demonstrate the efficacy of IPE training on improved decision-making in groups of mixed professional fields, which may enhance the ability of medical and nursing students to provide improved health care in the future.

One of the interesting findings of this study is that although the group work tasks were effectively performed by the mixed groups of students, about 16% of these groups made better decisions individually than as a group, potentially due to psychological barriers arising from issues related to traditional hierarchies among medical and nursing students. Indeed, traditional hierarchies and structures in the health care field may continue to create barriers that hamper effective interprofessional collaboration, [12] because aspiring health care professionals are traditionally not socialized in an interdisciplinary manner during their medical education. Our approach using a group discussion activity uncovered potential psychological barriers in medical and nursing students that could be overcome by early exposure to IPE training. We hope that this interdisciplinary interaction will break down hidden barriers and foster the development of effective collaboration among the participating students once they embark upon their professional careers. Furthermore, various factors, including team dynamics, collaboration skills, leadership, roles, and responsibilities, are known to contribute to the success of IPE [13,14]. Efforts to improve these factors, in addition to the mixing of students’ professional fields, may enhance their decision-making, which is a subject that needs to be evaluated in the future.

A limitation of this study is that there were substantial gender differences between the medical and nursing student groups. Indeed, of the 50 groups, 11 of the nursing student groups consisted of all female students and 3 medical student groups consisted of all male students. In contrast, all mixed groups included both male and female students, most likely as a result of the random assignment of the students. Therefore, it is possible that the positive effects of interdisciplinary groups on decision-making were, at least in part, due to the mixing of genders. Future research is needed to evaluate the effect of mixing student backgrounds, including gender and profession, on decision-making.
